# Editorial: Interplay of epigenetic regulation and cellular metabolism in cancer microenvironments

**DOI:** 10.3389/fimmu.2025.1735322

**Published:** 2025-11-13

**Authors:** Maria Virumbrales-Muñoz, Qihui Zhou

**Affiliations:** 1 Department of Obstetrics and Gynecology, University of Wisconsin-Madison, Madison, WI, United States; 2 School of Rehabilitation Science and Engineering, University of Health and Rehabilitation Sciences, Qingdao, China

**Keywords:** epigenetic, lactylation, diagnostic, metabolism, progression, diagnostics, biomarkers

The tumor microenvironment, an evolving and complex ecosystem where tumors develop, is highly influenced by tumor type, niche cell composition, and availability of biomolecules. Cancer cells have high and evolving energy demands, leading to broad changes in nutrient and metabolite availability, which forces cells to adapt to meet their proliferative and migratory needs. To survive, cells must adapt their metabolism in response to changes in the nutrients available. The outcome of this adaptation depends largely on the cell type: while cancer cells are highly plastic and can often adapt to using different nutrients, specific immune cells (e.g., NK and NK-T) are adversely impacted by the accumulation of specific metabolites (1) and others modify their phenotypes dramatically to adapt (e.g., macrophages, fibroblasts) (2).

In recent years, metabolic–epigenetic crosstalk has emerged as a central determinant of immune regulation and tumor progression. It is now well known that cell adaptation to different metabolic niches is enabled by epigenetic modifications that regulate gene expression and cell metabolism to support cell metabolic demands (3). At the same time, enzymes responsible for epigenetic modifications like histone methylation or acetylation require copious amounts of metabolites such as methionine or acetyl-CoA, respectively (4). Therefore, metabolite availability also influences the capability of chromatin remodeling enzymes to perform their regulatory functions. Thus, metabolite availability not only shapes the biochemical landscape but also directly governs the capacity of chromatin-remodeling machinery to execute regulatory functions.

This intricate interplay between metabolism and epigenetic control opens the door to new approaches in cancer diagnostics and therapeutics, a rapidly evolving area that is redefining how we view tumor biology and immune dysfunction. In this Research Topic of *Frontiers in Immunology*, we dissect the intersection between metabolism and epigenetics from multiple perspectives. The contributions span mechanistic studies of metabolic adaptation and chromatin regulation in tumor-infiltrating immune cells, systems-level analyses of metabolite-driven epigenetic reprogramming and include some highly novel translational insights into how metabolic checkpoint blockade might restore immune competence within tumors. Here we highlight a few of the most exciting contributions to the Research Topic.

In their review articles, Li et al. and Wang et al. discuss evidence that highlights the role of histone lactylation (i.e., addition of lactate groups to histones or DNA) as an important factor in metabolic reprogramming and epigenetic control in cancer. Such post-translational modification, fueled by the oncometabolite lactate, directly regulates gene expression by modifying histone lysine residues. Lactylation of histones or transcription factors has deep impacts on the TME. For example, Wang et al. describe evidence of histone modification that enhances protein translation efficiency and promotes tumor progression, a mechanism that is overrepresented in digestive tumors. Li et al.’s piece focuses on the role of lactylation as an energetic driver of tumor progression, supporting key cancer hallmarks like immune escape, angiogenesis, and metabolic flexibility; whereas the piece by Wang et al. explores the potential value of lactylation as a clinical prognostic tool. These novel insights are starting to show their potential therapeutic applications in pioneer studies demonstrating increased efficacy of anti-PD-1 therapy in combination with Lactate Dehydrogenase A inhibitors.

Circular RNAs (circRNAs) have rapidly become recognized as key post-transcriptional regulators that intersect with metabolic reprogramming, particularly in cancer. circRNAs regulate metabolism indirectly by controlling the expression of metabolic enzymes, transporters, and regulators of pathways like glycolysis, fatty acid oxidation, and glutaminolysis. The article by Shi et al. presented an atlas of the lung pre-metastatic niche circRNAs, identifying key dysregulations with normal tissue, including upregulation in key cellular elements such as myeloid-derived suppressor cells, macrophages, and alveolar epithelial cells, consistent with the dysregulation of recruitment-associated cytokines like S100A8/A9 and CXCL1/2. Dysregulated pathways included PI3K-Akt signaling, calcium signaling, neuroactive ligand-receptor interaction, and NF-κB signaling. Their findings were functionally validated via flow cytometry, thereby confirming their findings that lung epithelial cells inhibited apoptosis in the nascent niche, along with uncovering a potential role for the circRNA circRERE-PMN in regulating inflammation and apoptosis in the metastatic niche. Findings from these authors highlight the potential of circRNAs as early biomarkers for metastasis and potential therapeutic targets.

Shifting the focus to the role of germline genetics in susceptibility to cancer, Bi et al.’s work is a key piece of evidence bridging the gap between these fields. Their case-control study of 600 patients revealed a strong correlation of APOE gene polymorphisms with lung adenocarcinoma (LUAD). They concluded that the APOE ϵ2 allele was more common among patients with LUAD compared to normal controls, with increased risk with disease progression. The authors were able to identify patients with LUAD carrying the ϵ2 allele with highly abnormal lipid metabolism characterized by lower values for HDL, total cholesterol, and free fatty acids. The authors suggested that this metabolic dysregulation pointed toward a mechanism whereby the APOE ϵ2 allele creates a permissive metabolic environment for aggressive cancer development and progression.

Several papers highlighted the potential of metabolo-epigenomics as a diagnostic or prognostic tool. Among them, Yao et al. employed bioinformatics to investigate lncRNA (long non-coding RNA) networks in colorectal cancer. lncRNAs act as molecular scaffolds that recruit or sequester chromatin-modifying complexes to metabolic gene loci, thereby linking nutrient status to transcriptional regulation. The authors identified central hub genes MYC and STAT3 as dysregulated at the levels of transcription and translation in this cancer due to lncRNAs, (Yao et al.) and later corroborated their findings both *in silico* and *ex vivo* using database mining, qRT-PCR of clinical specimens, and immunohistochemistry. Their analysis indicated that both MYC and STAT3 exhibited robust diagnostic potential, highlighting their promise as clinical biomarkers for the early detection of colorectal cancers.

Finally, Chen et al. presented a compelling case for adding PAX1 gene methylation analysis to cervical cancer screening. Their clinical validation on a large patient cohort showed superiority of PAX1 methylation over conventional cytology to detect high-grade cervical lesions. They especially showed an improvement in the triage of patients with non-16/18 high-risk HPV infections, which would be an important improvement in a lacking clinical diagnostic space.

Collectively, the studies included in this Research Topic highlight how metabolic and epigenetic processes are intricately intertwined, influencing cancer cell behavior, immune adaptation, and clinical outcomes. These studies reveal that metabolism is not merely a bystander in gene regulation, but a fundamental driver of cellular identity and function. As the field advances, integrating multi-omics approaches and further validation of generated hypotheses will be essential to decode these interconnected networks and translate them into precision immunotherapies and metabolic interventions.
